# Signature molecules expressed differentially in a liver disease stage-specific manner by HIV-1 and HCV co-infection

**DOI:** 10.1371/journal.pone.0202524

**Published:** 2018-08-23

**Authors:** Amanda Whitmill, Seongcheol Kim, Vivian Rojas, Fahad Gulraiz, Kazi Afreen, Mamta Jain, Meharvan Singh, In-Woo Park

**Affiliations:** 1 Green Center for Reproductive Biology Sciences, Department of Obstetrics and Gynecology, University of Texas Southwestern Medical School, Dallas, Texas, United States of America; 2 Pharmacology and Neurosience, University of North Texas Health Science Center, Fort Worth, Texas, United States of America; 3 Microbiology, Immunology and Genetics University of North Texas Health Science Center, Fort Worth, Texas, United States of America; 4 Division of Infectious Disease, University of Texas Southwestern Medical School, Dallas, Texas, United States of America; Harvard Medical School, UNITED STATES

## Abstract

To elucidate HIV-1 co-infection-induced acceleration of HCV liver disease and identify stage-specific molecular signatures, we applied a new high-resolution molecular screen, the Affymetrix GeneChip Human Transcriptome Array (HTA2.0), to HCV-mono- and HIV/HCV-co-infected liver specimens from subjects with early and advanced disease. Out of 67,528 well-annotated genes, we have analyzed the functional and statistical significance of 75 and 28 genes expressed differentially between early and advanced stages of HCV mono- and HIV/HCV co-infected patient liver samples, respectively. We also evaluated the expression of 25 and 17 genes between early stages of mono- and co-infected liver tissues and between advanced stages of mono- and co-infected patient’s samples, respectively. Based on our analysis of fold-change in gene expression as a function of disease stage (i.e., early vs. advanced), coupled with consideration of the known relevant functions of these genes, we focused on four candidate genes, ACSL4, GNMT, IFI27, and miR122, which are expressed stage-specifically in HCV mono- and HIV-1/HCV co-infective liver disease and are known to play a pivotal role in regulating HCV-mediated hepatocellular carcinoma (HCC). Our qRT-PCR analysis of the four genes in patient liver specimens supported the microarray data. Protein products of each gene were detected in the endoplasmic reticulum (ER) where HCV replication takes place, and the genes' expression significantly altered replicability of HCV in the subgenomic replicon harboring regulatory genes of the JFH1 strain of HCV in Huh7.5.1. With respect to three well-known transferrable HIV-1 viral elements—Env, Nef, and Tat—Nef uniquely augmented replicon expression, while Tat, but not the others, substantially modulated expression of the candidate genes in hepatocytic cells. Combinatorial expression of these cellular and viral genes in the replicon cells further altered replicon expression. Taken together, these results showed that HIV-1 viral proteins can exacerbate liver pathology in the co-infected patients by disparate molecular mechanisms—directly or indirectly dysregulating HCV replication, even if lack of association of HCV load and end-stage liver disease in hemophilic patients were reported, and modulating expression of hepatocellular genes critical for disease progression. These findings also provide major insights into development of stage-specific hepatocellular biomarkers for improved diagnosis and prognosis of HCV-mediated liver disease.

## Introduction

Due to the shared routes of infection, HIV-1/HCV co-infection is common, with 15–30% of all HIV-1-infected persons estimated to be co-infected [1, 2]. HIV-1 co-infection is known to cause profoundly adverse consequences for HCV-mediated liver disease progression [3–6] by enhancing HCV replication and thereby increasing viral load in the infected patients [7, 8], even if lack of association of HCV load and end-stage liver disease in hemophilic patients were reported [9], and thus co-infection in Western countries has become a leading cause of morbidity and mortality in HIV-1-infected individuals [10, 11]. However, molecular mechanisms of this co-infected liver disease acceleration are still poorly understood, deterring development of therapeutics against HCV-mediated liver fibrosis.

One potential mechanism may occur through direct HIV-1 infection of HCV-infected hepatocytes followed by stimulation of HCV replication, resulting in expedited liver disease progression [7–9]. However, susceptibility of hepatocytes [12–15] and replicability of HIV-1 in hepatocytes are controversial [16–22]. Our previous data demonstrated that hepatocytes did not support HIV-1 infection [23]: Env-HIV-1 pseudovirus which successfully entered Jurkat cells failed to enter the hepatocytes, while VSVG-HIV succeeded, and even the transfected HIV-1 provirus expressed viral proteins but failed to generate infectious progeny virions in Huh7.5.1, suggesting that the observed enhancement of HCV replication and thus acceleration of liver disease progression in HIV-1 co-infected hosts were not due to the direct infection of HIV-1 into hepatocytes. Instead, transferable HIV-1 viral proteins, such as gp120 by shedding, Tat by diffusion, and/or Nef by exosomes/conduits, could trigger HCV-infected hepatocytes to exacerbate disease advancement. It is known that the shed Env glycoprotein (gp120) or gp120 associated with HIV-1 virions can directly interact with CXCR4 or CCR5 expressed on the surface of hepatocytes and transduce signals to alter HCV viral and HCV-infected cellular gene expression of hepatocytes [14, 15, 24]. HIV-1 Tat secreted from HIV-1 infected cells can diffuse into hepatocytes and augment HCV replication and certain hepatocellular genes to accelerate liver disease, as reported previously [25, 26]. HIV-1 Nef can be transferred from HIV-1-infected cells to the neighboring cells through conduits (filopodia) and/or exosomes [23, 27–30], and therefore the transferred protein in the target hepatocytes can dysregulate hepatocellular biology. However, it is unknown at present how HIV-1 and/or viral proteins induce these alterations in aggravating disease.

In addition, HIV-1 co-infection induces immunologic alterations, such as immune activation, apoptosis, and diminished HCV-specific T cell responses [31–34]. Specifically, HIV-1 activates the immune system by modulating the expression of cytokines, such as IL-4, -5, and -13, and TGFβ, critical for liver inflammation and fibrosis [33, 34] and enhances apoptosis of hepatocytes via a Fas/FasL and tumor necrosis factor-related apoptosis-inducing ligand (TRAIL) pathway [35]. Furthermore, co-infection induces accumulation of cytotoxic CD8 T cells in the liver, which increases inflammatory mediators, leading to increased liver tissue damage in the co-infected patients [34, 36–38]. However, the molecular details and significance of these HIV-1 co-infection-associated immunologic changes in expedited liver disease progression are largely unknown.

Whatever the molecular mechanism might be and whichever HIV-1 viral elements are the most responsible elements for accelerating liver disease progression, the factors and processes lead to dysregulation of hepatocellular gene expression and/or HCV replication, critical for accelerating co-infected liver malady. We accordingly performed mRNA microarray analysis with the Affymetrix GeneChip Array, containing 67,528 well-annotated genes, to identify differentially expressed genes from HIV-1 co-infection within early vs. advanced fibrosis. We identified numerous genes, whose expression was specifically modulated by the presence of fibrosis in the co-infected patients, establishing that the strategy can provide vital information on how HIV-1 co-infection modulates HCV replication and liver disease-related gene expression in HCV-infected hosts, and on the potential of those genes for development as biomarkers in diagnosis and prognosis of hepatic disease progression in the co-infected patients.

## Materials and methods

### Ethics statement

The protocol employed for this study was reviewed and approved by the Institutional Review Board at all of the participating sites, and written informed consent for use of the liver biopsy samples in research was obtained from each patient. All human subjects were adult.

### Human subjects

Twelve patients (3 patients in each of early and advanced stages of liver diseases who were HCV-mono- and HIV/HCV co-infected) who underwent a liver biopsy as standard of care for HCV staging were recruited from the clinic at University of Texas Southwestern Medical Center (UTSWMC). The protocol employed for this study was reviewed and approved by the Institutional Review Board at all of the participating sites, and written informed consent for use of the liver biopsy samples in research was obtained from each patient. Infection of HCV was confirmed by a positive RNA polymerase viral load, and liver fibrosis stages for HCV patients were assessed from biopsy (Batts-Ludwig) or ultrasound reports in patient records from the time of biopsy: early (F1, portal fibrosis and F2, periportal fibrosis with rare bridges) and advanced (F3, bridging/septal fibrosis and F4, cirrhosis) stage [39]. Infection and viral load of HIV-1 in the patients were determined by a combination of ELISA (enzyme-linked immunosorbent assay) with Western blot analysis for the viral antigen, and all co-infected patients were under treatment of combination anti-retroviral therapy (cART). At the time of liver biopsy, 2–3 mm of liver tissue was placed in RNALater (Life Technologies, Mountain View, CA) at 4^0^ C overnight and then stored in liquid nitrogen.

### Transcriptome array

Total RNA was isolated from the liver biopsy samples, using the RNeasy kit (Invitrogen life technologies, Carlsbad, CA), according to the manufacturer’s protocol, and a new high-resolution array from Affymetrix, GeneChip Human Transcriptome Array 2.0 (HTA2.0), was undertaken at the UTSWMC core facility to interrogate all transcript isoforms in the human transcriptome with 6 million probes targeting coding transcripts, exon-exon splice junctions, and non-coding transcripts to identify molecular signatures in each of the early and advanced stages of liver disease from HCV mono- and HIV-1/HCV-co-infected liver samples. To evaluate the microarray data, we employed one-way between-subject ANOVA (unpaired) and ANOVA p-value (condition pair) analysis wherein a p-value of < 0.05 was considered statistically significant (*). Fold change (linear) <-2 or fold change (linear) >2 was used as default filter criteria.

### Cells, plasmids, and viruses

**Cells**: Huh7.5.1 and its subgenomic replicon expressing regulatory genes of the JFH1 strain of HCV with Renilla luciferase as a reporter gene in Huh7.5.1 (APC140), were obtained from Apath, L.L.C. (New York, NY). Both cell lines were cultured in DMEM/10% fetal bovine serum in the presence (APC140) or absence (Huh7.5.1) of 0.5 g/L G418 (Geneticin, ThermoFisher Scientific, Waltham, MA). **Plasmids**: The full length of the nef or tat open reading frame was placed under CMV promoter and tagged with Myc epitope at its 3’ end. Plasmids expressing acyl-CoA synthetase long-chain family member 4 (ACSL4), glycine N-methyltransferase (GNMT), and interferon alpha inducible protein 27 (IFI27) were purchased from OriGene Technologies (Rockville, MD). *ACSL4* and *IFI1* genes were tagged with Myc epitope. **Viruses**: Pseudotype viruses were generated, as described previously [23]. Wild type- (wt-) and nef-deficient-viruses (Δnef-HIV-1) were produced by transfecting their corresponding proviruses into Jurkat clone E6-1 (Jurkat T cells) which was obtained through the NIH AIDS Reagent Program [40], and the purified virions of HIV-1 or Δnef-HIV-1 were obtained by precipitating viruses from the culture supernatants in 20% sucrose cushions. Purified virions hence contained only virus particles, not culture supernatants, so as to exclude effects of soluble molecules, appearing in those supernatants. Virus levels were quantified by measuring viral reverse transcriptase activity [23].

### Reverse transcriptase assay

One ml of the culture supernatant—from the transduced HEK-293T (293T) cells or from Jurkat T cells transfected (or infected) with the indicated amount of proviral DNAs (or viruses)—was collected, virions in the supernatant were pelleted by centrifugation at 12,000 g for 1 hr, and the RT activity was determined, as described [41].

### Transfection of plasmids and HCV JFH1 genomic RNA

Huh7.5.1 or APC140 was transfected with the indicated plasmids, using lipofectamine 3000 (Thermofisher), as described previously [23]. To treat these cells with HIV-1 Env, the cells were incubated with the indicated inoculum of psuedotype viruses (VSV-G or VSV-Env), HIV-1, or purified HIV-1, not with the synthetic Env, for 2 days and lysed with an appropriate lysis buffer for qRT-PCR, Luciferase assay, or Western blot analysis. For the transfection of the full-length HCV JFH1 RNA, the pFL-JFH1 plasmid was linearized with XbaI, purified, and transcribed with MEGAscript^TM^ T7 RNA transcription kit (Ambion, Austin, TX). The RNA transcripts were treated with 2 U DNase I at 37^0^ C for 30 min, purified by acid phenol/chloroform extraction and suspended in diethylpyrocarbonate-treated water. The transcribed RNA was then transfected, using lipofectamine 3000 (Thermofisher), as described [23].

### Quantitative real-time polymerase chain reaction (qRT-PCR)

RNAs were isolated from liver biopsy samples for Transcriptome array, Huh7.5.1, or subgenomic replicon (APC140, Apath), using the RNeasy Micro Kit (Qiagen, Germantown, MD), and the RNAs were quantified by the VeriQuest SYBR Green One-Step qRT-PCR Assay (Bio-Rad, Hercules, CA), using the indicated primer pairs of each gene. The β-Actin house-keeping gene was used as an internal control for qRT-PCR, and U6-2 [Applied Biological Materials (ABM), Canada] was employed as an internal control for miR122. Melting curve analysis was performed to confirm the specificity and purity of the amplified product.

### Reporter gene assay

The Luciferase reporter gene assay was performed, using the Renilla Luciferase assay kit (Promega, Madison, WI), according to the manufacturer’s protocol. Briefly, HCV subgenomic replicon cells, APC140, treated with HIV-1 pseudovirus or virions or transfected with the indicated plasmids for 48 hr, were washed twice with ice-cold PBS and lysed in Renilla luciferase lysis buffer. The luciferase activity in the lysates was determined with a luminometer (Promega) in triplicates of three independent experiments.

### Western blot analysis

Western blot analysis was performed, as described (26). The primary antibodies employed for the analyses were β-actin (A1978, Sigma, St. Louis, MO), Myc (Santa Cruz, Dallas, TX) for Tat, Nef, ACSL4 and IFI27 (Santa Cruz), NS5A and GNMT (Santa Cruz). The horseradish peroxidase (HRP)-conjugated secondary antibodies and the enhanced chemiluminescence (ECL) were purchased from ThermoFisher and Bio-Rad, respectively.

### Confocal analysis

Huh7.5.1 and APC140 cells grown on polylysine-coated cover slips were transfected with the indicated plasmids and cultured for 48 hr. To label the endoplasmic reticulum (ER), pDsRed2-ER (Clontech, Mountain View, CA) was transfected into the cells. Cells were then washed twice with PBS, fixed in 4% paraformaldehyde, permeabilized with 0.3% Triton X-100 for 5 min, and blocked in 2.5% BSA for 30 min. Cells were incubated with the primary antibody followed by Alexa Fluor 488-conjugated to secondary antibodies at room temperature for 1 hr each and washed three times with PBS to visualize subcellular localization of the indicated proteins with a confocal microscope, as described [42].

### Data analysis

All values are expressed as means ± SD of triplicate experiments. All comparisons were made based on the control using a two-tailed Student’s *t*-test. A *p* value of < 0.05 was considered statistically significant (*), and *p* < 0.001 highly significant (**).

## Results

### Characteristics of HIV- and/or HCV-infected subjects

Clinical characteristics of the subjects are shown in Table 1.

**Table 1:** Characteristics of HIV-1 and/or HCV-infected subjects.

Sample ID	HIV/HCV status (Liver Dis. Stage)	Total Chol.	LDL	HDL	ALT	AST	CD4	HIV Viral Load	HIV Medications	Cholesterol Lowering Medications	Notes
KV1	HCV (0–2)	164 +/-6.7	78 +/-25.1	52 +/-23.4	40	36	NA	NA	NA	NA	
KV2					16	29	NA	NA	NA	NA	
KV3					16	28	NA	NA	NA	NA	
KV4	HCV (2–4)	150 +/-60.4	101 +/-46.5	25 +/-11.8	14	24	NA	NA	NA	NA	
KV5					192	171	NA	NA	NA	NA	
KV6					31	30	NA	NA	NA	NA	
KV7	HIV/ HCV (0–2)	182 +/-30.6	88 +/-28.7	48 +/-19.0	81	103	728	ND	Truvada/ Raltegravir	NA	
KV8					22	27	740	52	Truvada/ Raltegravir	NA	
KV9					19	27	1034	ND	Avacavir/ Dolutegravir/Lamivudine	NA	HC/ Endo. HG
KV10	HIV/ HCV (2–4)	156 +/-46.0	85 +/-53.1	37 +/-11.9	12	18	691	ND	Ritonavir	Atorvastatin/Fish oil	
KV11					156	258	313	9273	Nelfinavir/ Avacavir/ Lamivudine	Flaxseed oil	HL/ mixed HC/HT
KV12					36	82	289	65	Effavirenz/ Epzicom	NA	

NA and ND indicate “Not Available” and “Not Detected”, and Chol, HC, HG, HL, and HT stand for cholesterol, hypercholesterolemia, hyperglyceridemia, hyperlipidemia, and hypertriglyceridemia, respectively.

In total, 12 patients (KV1-12) were recruited in this study—3 patients in each of early (0–2) (KV1-3 and KV7-9) and advanced (3–4) stages (KV4-6 and KV10-12) of liver diseases who were HCV-mono- (KV1-6) and HIV/HCV co-infected (KV7-12), respectively. Liver disease stages of each patient were assessed based on who underwent a liver biopsy as standard of care for HCV staging, where all HIV-1-infected patients were receiving combinatorial anti-retroviral therapy (cART). Notably, values of aspartate aminotransferase (AST) and alanine aminotransferase (ALT) in all but KV5 (advanced stage of HCV mono-infected) and KV7 and KV11 (early and advanced stage of HIV/HCV-co-infected, respectively) patients fell within normal ranges, and viremia was detected in the periphery of a few co-infected patients (KK8, KV11, and KV12), where the number of CD4 cells in KV11 and KV12 was below 350/ml, even under the cART treatment. However, we did not perform covariate or multivariate analyses to find associations among gene expression, CD4 count, HIV viral loads, and the use of antiretroviral therapy, since each patient subgroup was small for statistically robust correlation analysis.

### Liver disease stage-specific common transcriptional signatures of HIV- and/or HCV-infected patients

**Fig 1:**
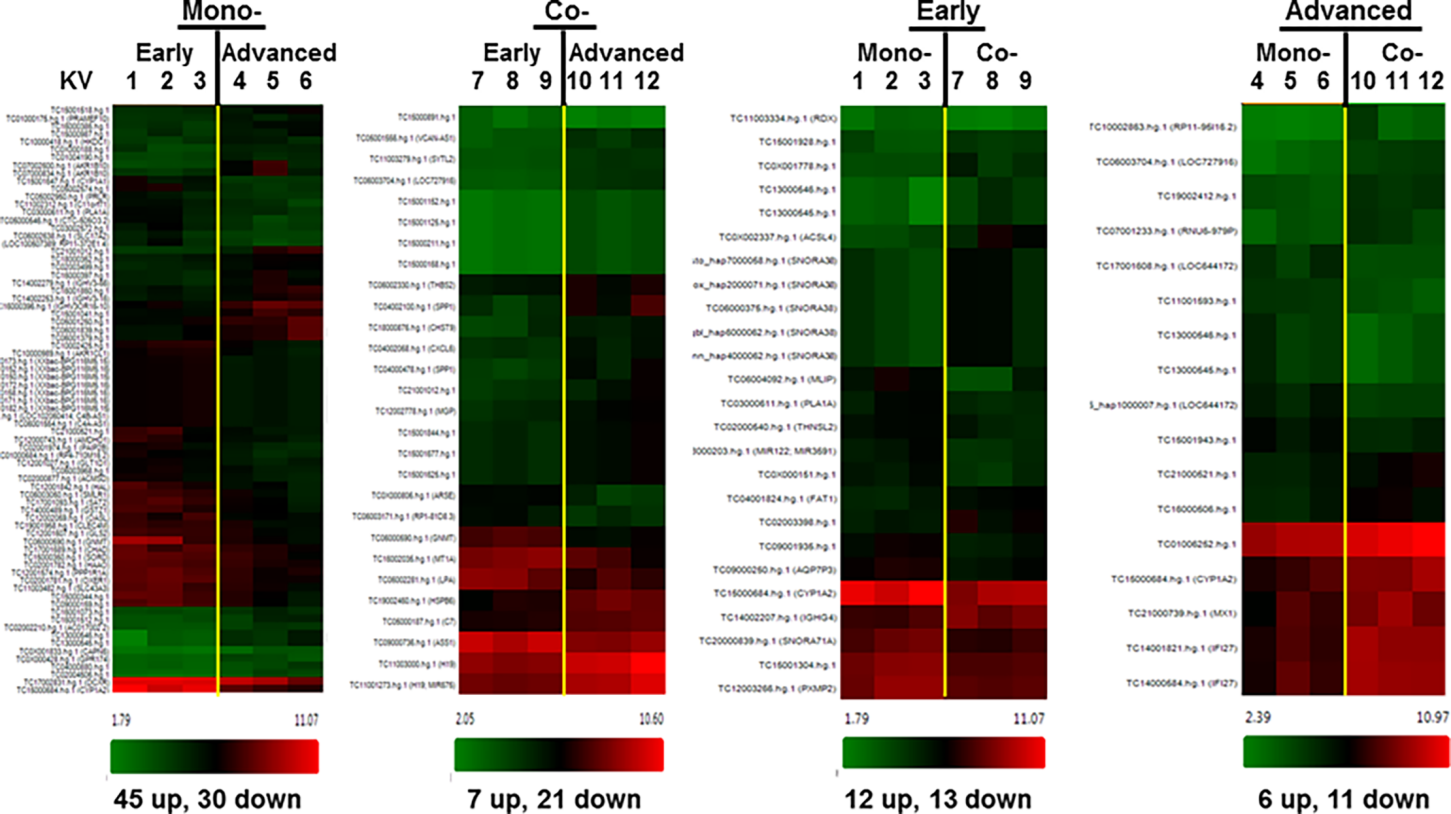
Heatmap generated from the microarray data. Mono- and Co- indicate HCV mono- and HIV/HCV co-infection, respectively, and below shows the number of genes up- or down-regulated by the indicated stages of liver disease by HCV mono- or HIV/HCV co-infection. Numbers of KV above the heatmap represent sample ID in the Table 1.

**Fig 2:**
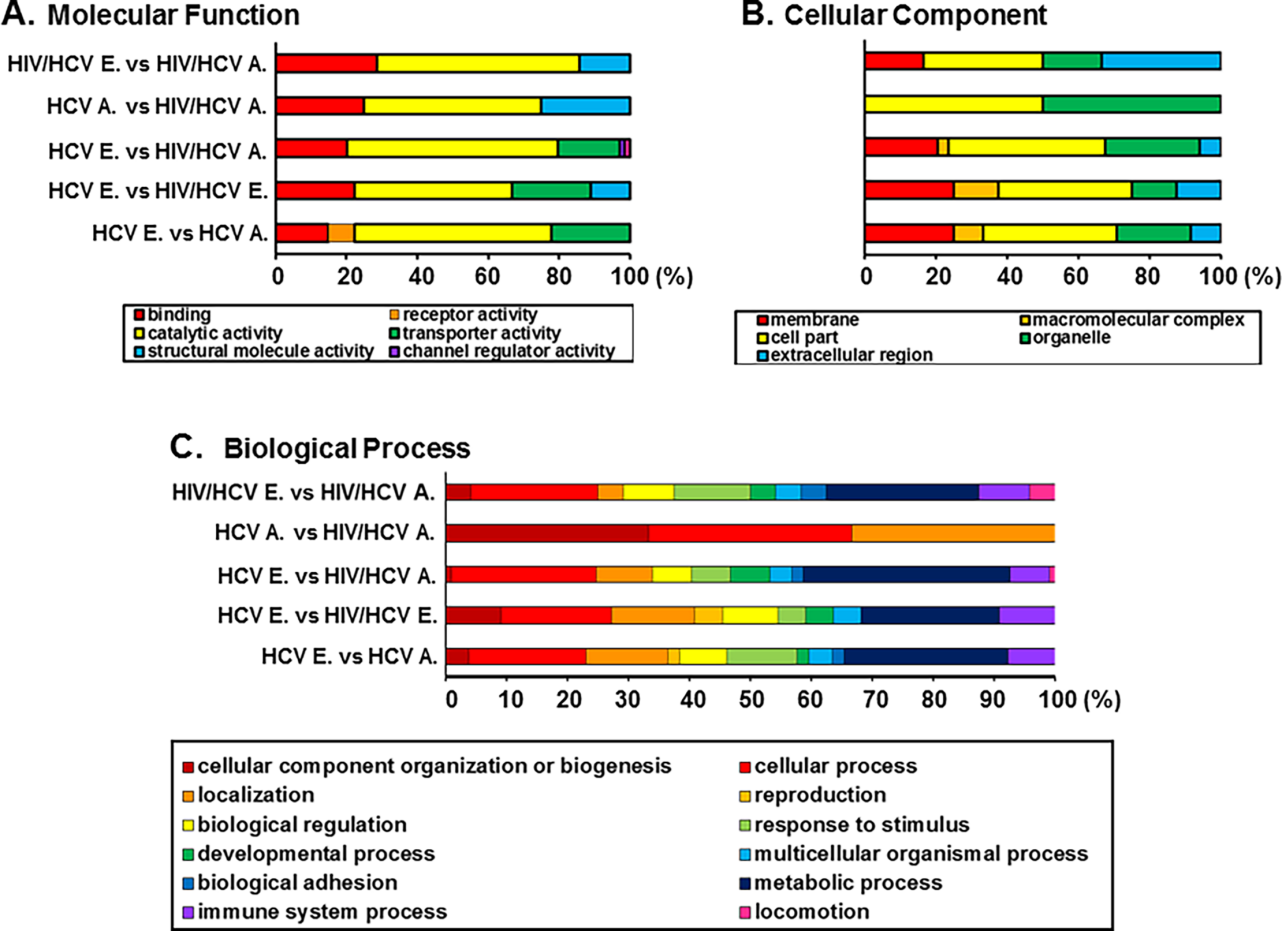
Panther analysis of the differentially expressed cellular genes. Panther (http://pantherdb.org) analysis for Molecular functions of genes from each comparison (A), cellular compartment (B), and biological processes associated with genes from each comparison (C).

To understand molecular mechanisms for how HIV-1 co-infection expedites HCV-mediated liver disease advancement and to identify a molecular signature common to the stage of liver disease from mono- (HCV only) or co- (HIV/HCV) infected patients, we performed a new high-resolution assay from Affymetrix, GeneChip Human Transcriptome Array (HTA2.0), using the early and advanced stages of liver disease from HCV-mono- and HIV/HCV-co-infected liver specimens. Employing a two-way ANOVA approach, out of 67,528 well-annotated genes, we identified 75 (45 up- and 30 down-regulated) and 28 (7 up- and 21 down-regulated) genes expressed differentially between early and advanced stages of HCV mono- (mono-, Fig 1) and HIV/HCV co-infected (Co-, Fig 1) patient liver samples, respectively, and 25 (12 up- and 13 down-regulated) and 17 (6 up- and 11 down-regulated) genes between early stages of mono- and co-infected liver tissues and between advanced stages of mono- and co-infected patient’s samples, respectively, as shown in the Heatmap (Fig 1). To obtain a more basic output array analysis, the Affymetrix Transcriptome Analysis Console (TAC) software was first employed, and Pathway analysis supported by the TAC software, DAVID [43, 44], PANTHER [45, 46], and WIKI Pathways Beta [47] was used to conduct more elaborate data analysis. Analysis based on the molecular functions showed that the genes encoding proteins with catalytic activity were the most abundant (yellow in Fig 2A), regardless of disease stage or mono-/co-infection. The gene products were distributed among diverse compartments of liver cells, while 50% of them were detected in the cytosol (yellow) and the rest of them were localized to the subcellular organelle, when disease reached the advanced stage from mono- and co-infection (Fig 2B). Similar dramatic changes in biological processes were detected at the advance stages of mono- and co-infection; that is, the differentially expressed gene products were involved in the regulation of various biological processes at the different stages of liver disease. However, when the liver complications had reached advanced stages, the genes involved in metabolic processes (dark blue, Fig 2) completely vanished, while only 3 different types of genes, functioning in biogenesis (dark red), cellular processes (red), and subcellular localization (brown) were differentially expressed (Fig 2C). Taken together, these data indicated that numerous hepatocellular genes were differentially expressed in a liver disease stage-specific manner by mono- and co-infection and that the most attractive genes among them are encoding proteins with enzymatic activities involved in metabolic processes in the cytosol or subcellular organelle.

### Molecular signatures expressing differentially in a disease stage-specific manner

**Fig 3:**
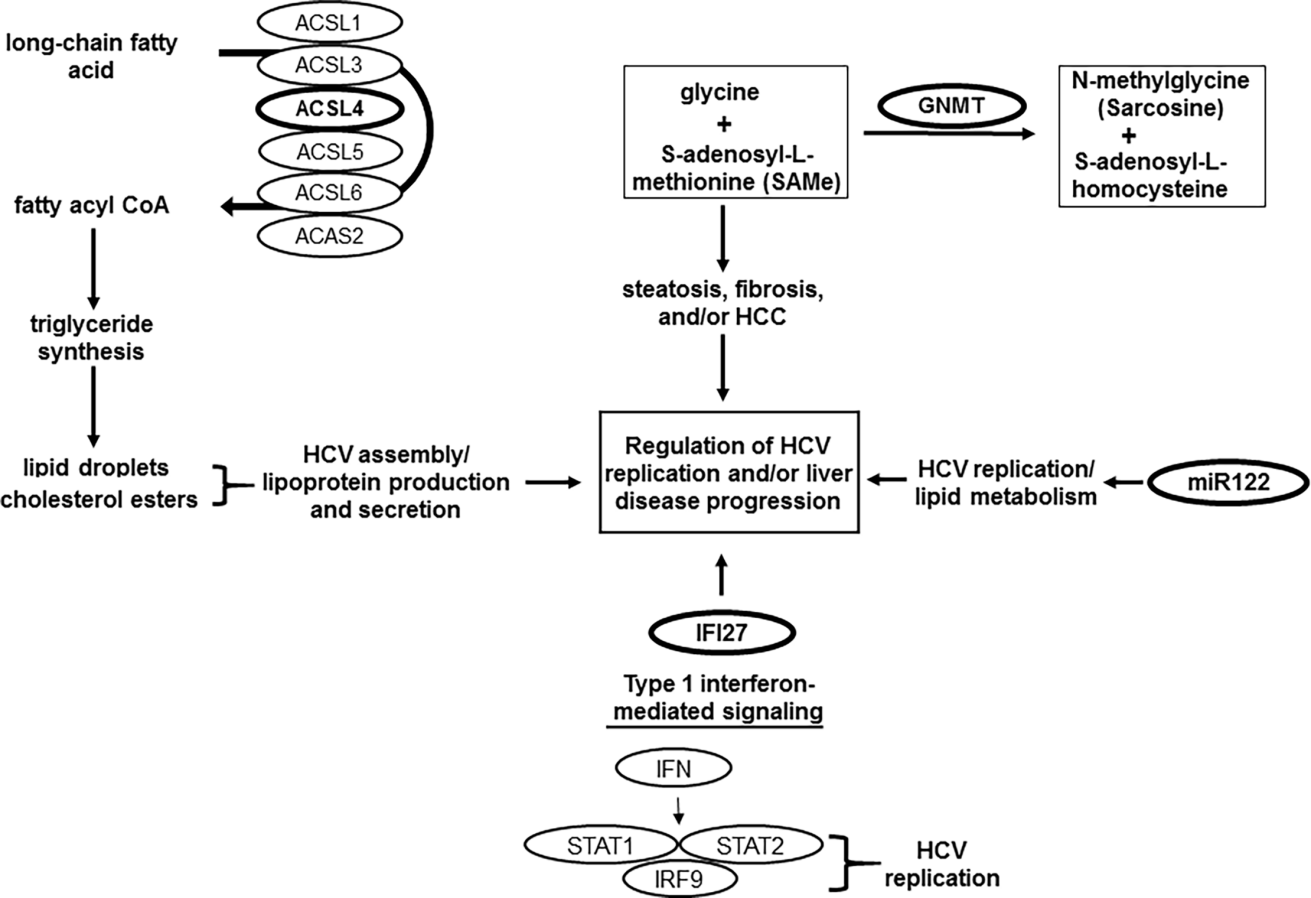
Functions of ACSL4, GNMT, IFI27, and miR122. The 4 genes that we have chosen are shown as bold, and other related critical genes are shown as plain. Molecular processes of each gene leading to regulation of HCV replication and/or liver disease progression are described, and references on the function are cited in the text.

**Fig 4:**
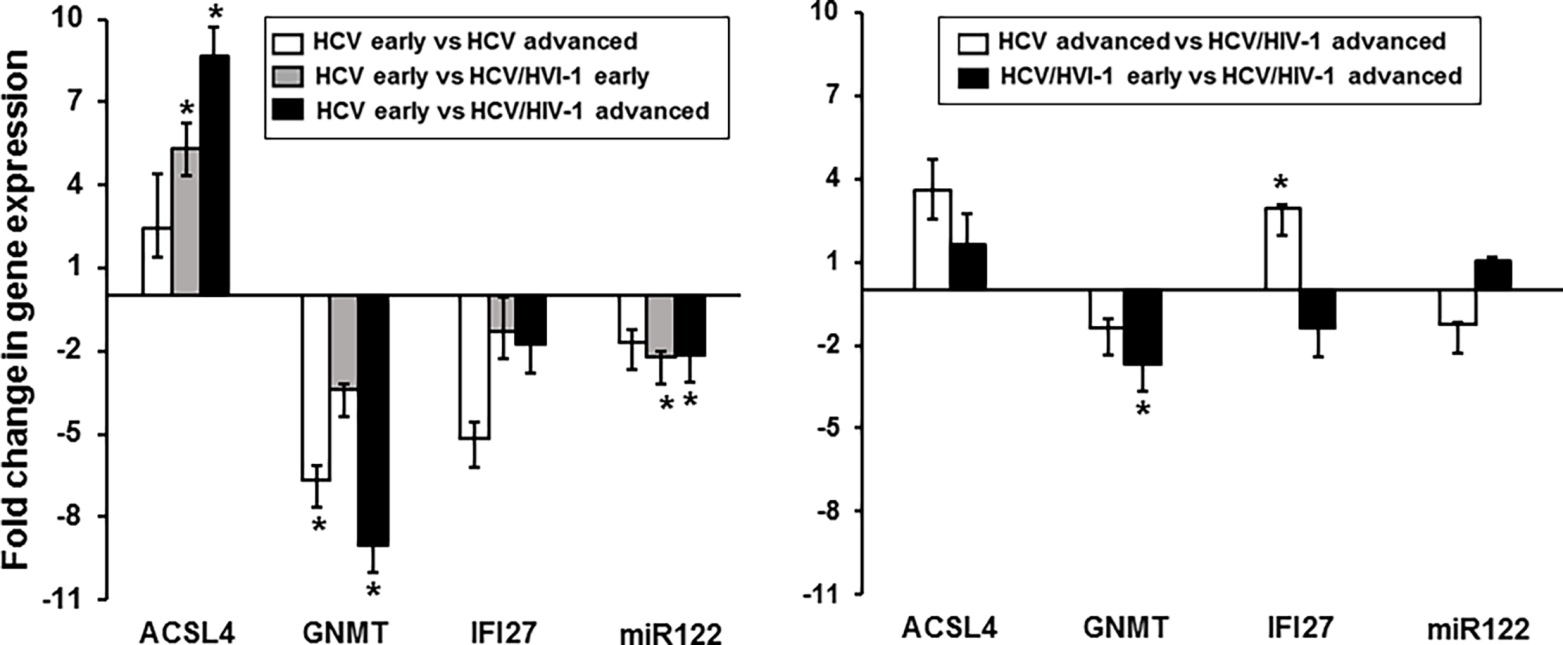
PANTHER analysis for big picture summary of genes—ACSL4, GNMT, IFI27, and miR122. Fold changes of each gene associated with disease progression by mono- and co-infection were depicted, and the plugged genes from array comparisons that met 2 fold change and 0.05 p-value were presented.

Next, we further analyzed the statistical significance of the differentially expressing genes in a disease stage-specific manner by co-infection with the PANTHER analysis (http://pantherdb.org) (42,43), keeping the above functional analyses in mind. Focusing on the above noted functions, and on the statistical significance of the differential expression/disease-stage associations, we have chosen the following 4 genes—*ACSL4*, *GNMT*, *IFI27*, and *miR122*—for further study. First, as shown in Fig 3, the *ACSL* family comprises many isoforms, *ACSL1*, *3*, *4*, *5*, *6*, and *ACAS2* (Acetyl-coenzyme A synthetase), which are splice variants or have different initiation sites for translation [41], converting long-chain fatty acid (FA) to fatty acyl CoA [41] for lipid synthesis or fatty acid oxidation [41, 43–45]. The lipid molecules are critical sources for the formation of lipid droplets, which play a vital role in HCV replication and assembly [22, 48–50], as summarized in Fig 3. Interestingly, our transcriptome array indicated that HVI-1 co-infection up-regulated only *ACSL4*, but not other isoforms, in a liver-disease stage specific manner (Fig 4). Insofar as up-regulation of genes encoding enzymes critical for the synthesis of FAs and complex lipids is one hallmark of tumorigenesis [46, 47], and up-regulation of *ACSL4* is particularly linked to hepatocellular carcinoma and other aggressive cancers [51–54], our finding of differential expression of *ACSL4* points to the significance of the gene in regulation of hepatic disease progression in a disease stage-specific fashion in the co-infected patients.

In contrast to *ACSL4*, expression of *GNMT* was significantly downregulated by co-infection; that is, our transcriptome analysis revealed that expression of *GNMT* was reduced by approximately 3 fold, and further reduced by 7- and 9-fold at the advanced stage of mono- and co-infection, respectively, compared with the early stage of HCV mono-infection (Fig 4). It is reported that GNMT, the most abundant methyltransferase in the liver [55], has tumor suppressor function [56] and is associated with nonalcoholic fatty liver disease (NAFLD) which accompanies hepatic methionine deficiency and homocysteine elevation [57–59] (Fig 3). Thus, the significant decline of GNMT in a stage-specific fashion by mono- and co-infection suggests that GNMT plays a key role in acceleration of progression of HCC.

IFI27, an interferon alpha-inducible protein, involved in the IFN pathway [60–66], was found to be up-regulated in certain cancers, including HCC [67–69]. Recent results showed that expression of *IFI27* was up-regulated in HCV-infected hepatocytes and HCV-related chronic liver disease (CLD), but not in HBV-related CLD or HCC [68], specifically indicating that IFI27 is integral to HCV-mediated HCC. Further, the levels of *IFN27* and other IFN-stimulated genes (*ISG*) are elevated by HCV infection through activation of the type I IFN-induced Jak/STAT signaling pathway [70, 71] (Fig 3). Our microarray data revealed that *IFI27* was increased approximately 3 fold at the advanced, rather than the early, stage of liver disease by co-infection, which is statistically significant (Fig 4), and the observed differential expression of IFN-related genes by co-infection is consistent with other studies [36, 72–76], showing the importance of the gene in accelerating liver malady in co-infected patients.

*miR122* is a liver-specific microRNA (*miRNA*) [77, 78], which promotes the HCV life cycle by binding to the 5’ untranslated region (UTR) [79, 80] and modulates expression of mRNAs involved in cholesterol biosynthesis, proliferation, and cell differentiation [81–84], as depicted in Fig 3. Expression of *miR122* is typically reduced or lost in liver cancers, while maintained in HCV-associated liver cancer [85, 86]. Our microarray data with patients’ liver samples indicated that the level of *miR122* declined in the co-infected patients, a statistically significant change (Fig 4), showing that this reduction results from HIV-1 infection. However, the molecular mechanism of *miR122* action, and whether it is essential for liver disease acceleration in the co-infected patients are unclear.

Taken together, these data and the previous reports suggest that the four selected genes are essential candidates for stage-specific signature molecules of the advancing hepatic disease; elucidation of the relationship between differential expression of these genes and clinical liver disease stages will bring insights into the development of diagnostic/prognostic biomarkers to evaluate stage-specific liver disease progression.

### qRT-PCR analysis of the genes to confirm the integrity of microarray data

**Fig 5:**
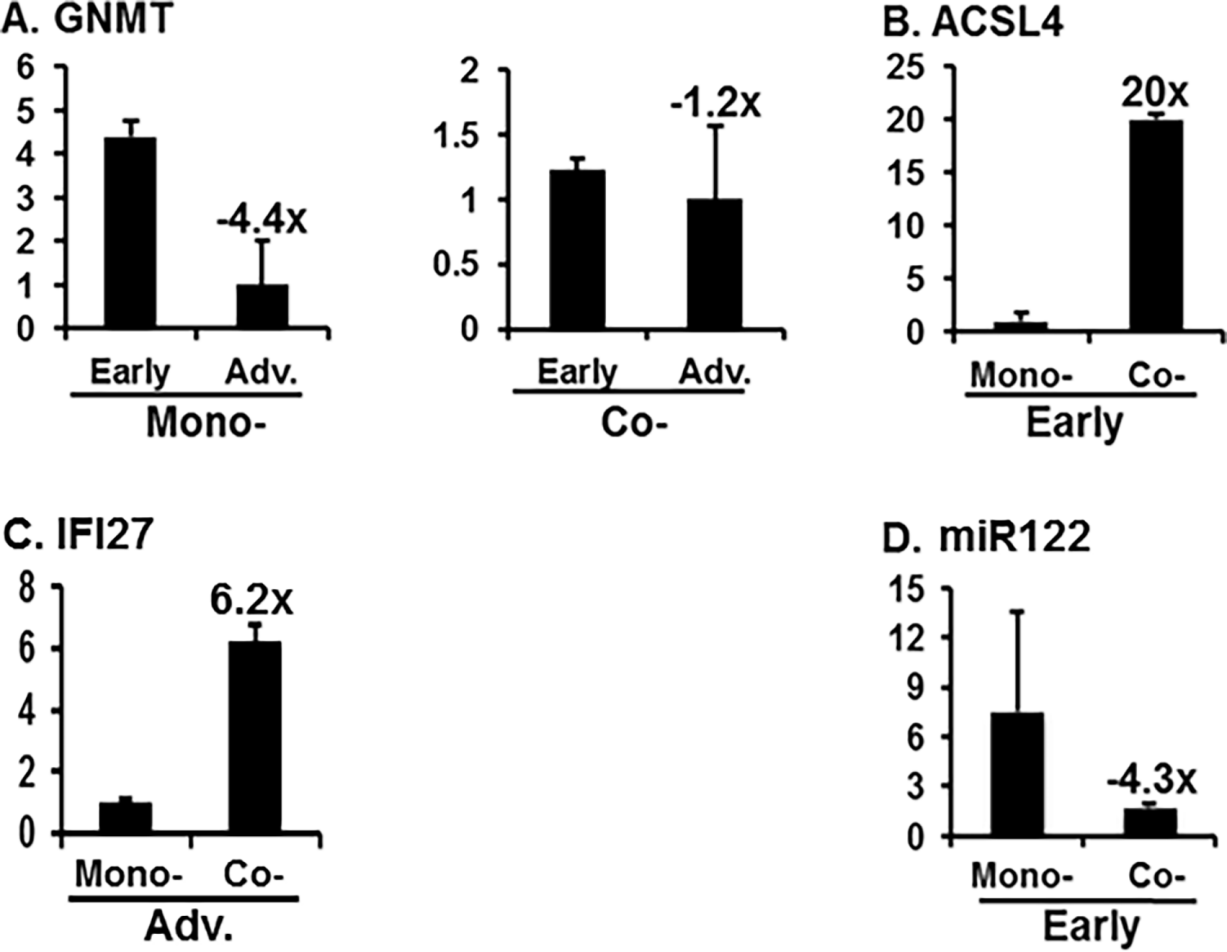
Confirmation of integrity of the microarray data qRT-PCR analysis. RNAs isolated from the indicated stages of 3 of each mono- and co-infected patients’ liver samples were quantified by the VeriQuest SYBR Green One-Step qRT-PCR Assay (Bio-Rad, Hercules, CA), as triplicate, using the specific primer pairs of each gene. β-Actin house-keeping gene was used as an internal control for qRT-PCR and U6-2 [Applied Biological Materials (ABM), Canada] was employed as an internal control for miR122. Melting curve analysis was done to confirm the specificity and purity of the amplified product.

We then sought to confirm the integrity of differential expression of the genes, whose expression was deemed statistically significant by qRT-PCR with RNAs isolated from the indicated stages of mono- and co-infected patients’ liver samples. Our data indicated that each gene expression pattern was essentially unchanged vs that of the microarray analysis, although the fold changes derived from the microarray data were not precisely identical to those of the qRT-PCR. Specifically, qRT-PCR analysis indicated that *ACSL4* was augmented 20 fold (Fig 5B), although the change was higher than the increase in microarray data (5.3 fold), at early-stage co-infection, compared with mono-infection in Fig 4. The amount of *GNMT* was reduced from the early to late stage of liver disease by 6.6 fold and by 9 fold in the mono- and co-infected patients, respectively (Fig 4). In corroboration, a similar pattern of reduction (4.4 and 1.2 fold, respectively) was observed in the qRT-PCR, as shown in Fig 5. Our qRT-PCR analysis with the patient samples showed that expression of *IFI27* was elevated 6.2 fold at the advanced stage of co-infection, compared with the same stage of mono-infection (Fig 5), which was again consistent with the microarray data, wherein the increase was approximately 3 fold (Fig 4). The *miR122* level was reduced 2.2 fold (Fig 4) and 4.3 fold (Fig 5) in the microarray analyses and in the qRT-PCR, respectively, with the same stages of mono- and co-infected patients’ RNA samples. Taken together, these data confirmed the integrity of the microarray analysis, though the fold changes were not identical, and showed that HIV-1 co-infection plays an essential role in regulating transcription of hepatocytic genes in a disease stage-specific way.

### Subcellular localization of ACSL4, GNMT, and IFI27

**Fig 6:**
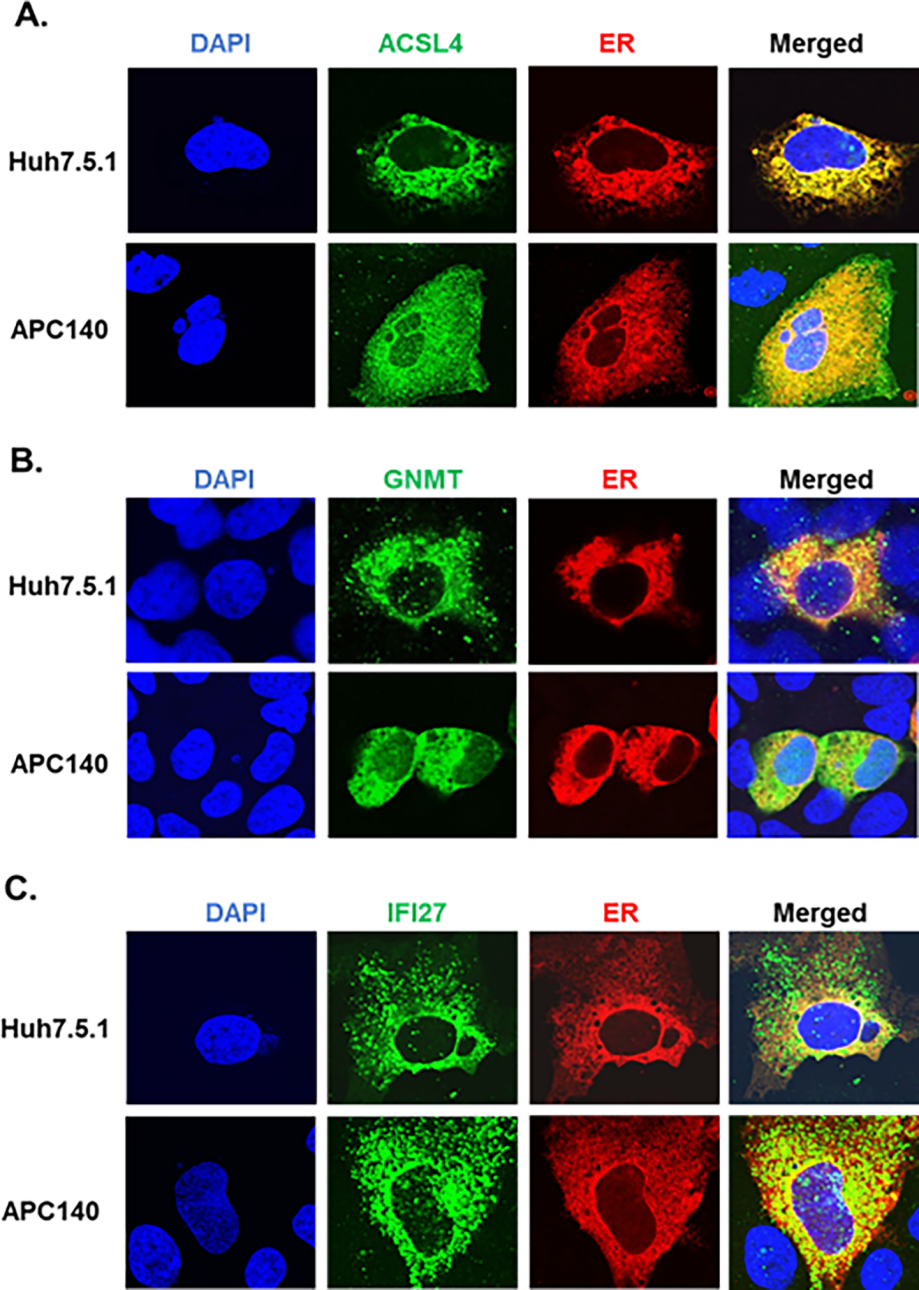
Confocal microscopy. Subcellular localization was determined by confocal microscopic analysis after transfecting the indicated genes together with pDsRed2-ER (Clontech) into Huh7.5.1 and its replicon cells, APC140. Expression of each protein was detected by anti-Myc Ab for ACSL4 (A) and IFI27 (C) and by anti-GNMT Ab for GNMT (B) followed by Alexa Fluor 488. Thus, expression of the cellular genes shows green color, and expression of pDsRed2-ER encodes a fusion consisting of red fluorescence protein, showing red color in the endoplasmic reticulum (ER) of the transfected cells; DAPI depicts the nucleus.

To determine the role of these genes in HCV replication, we first investigated the possibility of ER localization of the expressed proteins, since it is very well established that HIV-1 co-infection upregulates HCV replication which takes place in the lipid-rich subcellular milieu of ER [87–90]. Accordingly, we co-transfected plasmid expressing ER marker protein with each of the genes encoding ACSL4, GNMT, or IFI27 into Huh7.5.1 and APC140, and confocal microscopic analysis was performed. As shown in Fig 6A, ACSL4 was expressed in the cytoplasm (green color), and the ER marker was detected in the ER (red color), as expected. When the two images were merged, a significant portion of the ACSL4 in both Huh7.5.1 and APC140 was detected as yellow color in the ER, where the ER marker was expressed (red in Fig 6A). Similar results were obtained with GNMT (Fig 6B) and IFI27 (Fig 6C), indicating that all these proteins are distributed to the cytoplasm and cytoplasmic organelle, ER. Localization of these proteins to the ER also implies that these proteins could play important roles in regulating HCV replication.

### Impact of the selected cellular genes on HCV replication

**Fig 7:**
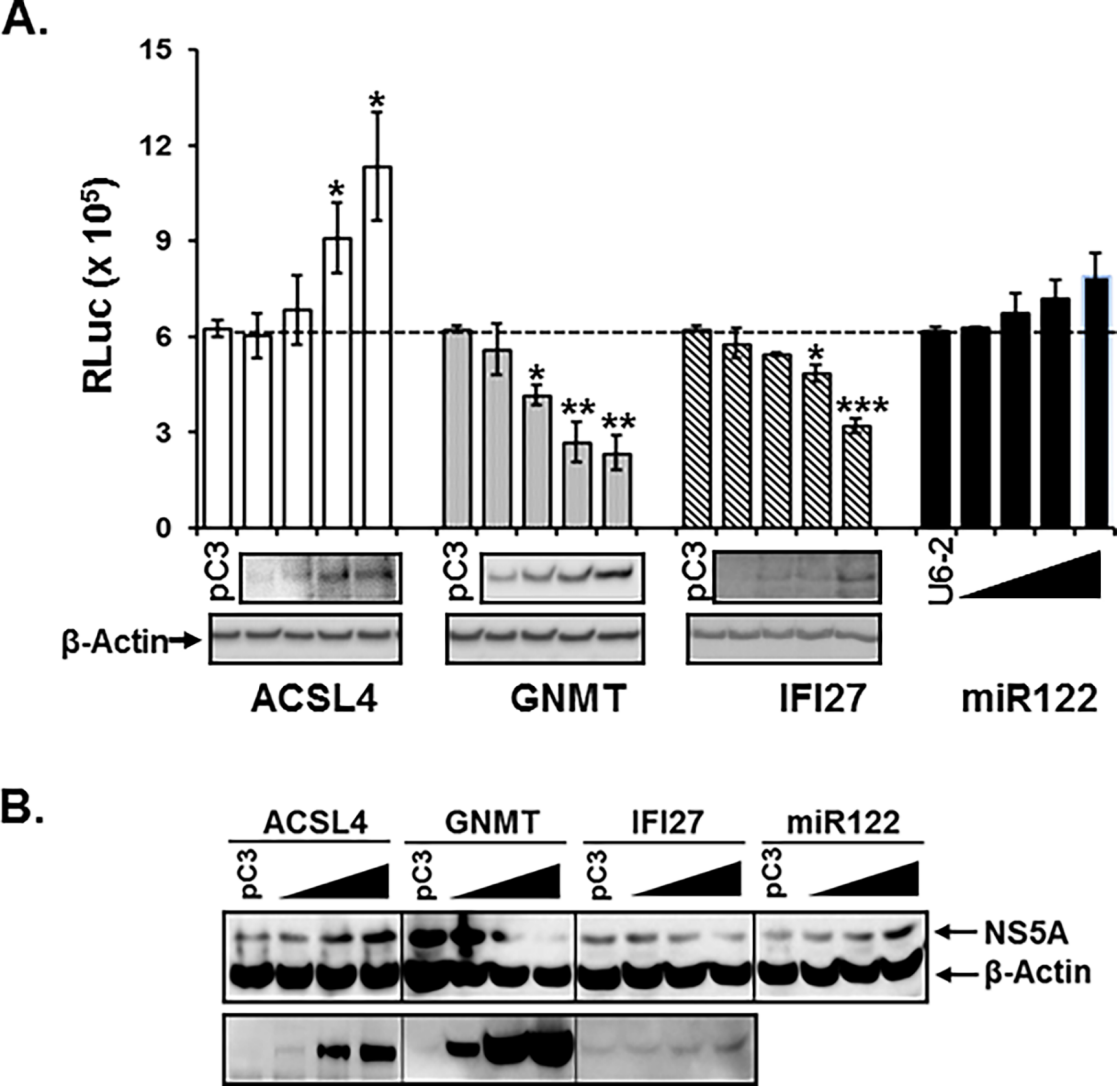
Impact of ACSL4, GNMT, IFI27, and miR122 on HCV replication. **(A)** APC140 was transfected with isotype plasmid (pC3) together with 0.2, 0.4, 0.8, and 1.6 μg of plasmid expressing each gene or 12.5, 25, 50, and 100 nM of *miR122* by lipofectamine method, and at 48 h post-transfection, cells were lysed with lysis buffer for Renillar luciferase assay (Promega); lysates were used to measure Renillar luciferase activity (bar graph) and to analyze expression of the indicated proteins by WB analysis (low). For *mir122*, *U6-2* was employed as an internal control. **(B)** Huh7.5.1 in 6 well plate was first transfected with 2 μg of HCV JFH1 RNA and 6 h after RNA transfection, with 0, 1, 2, and 4 μg of the indicated plasmid expressing each gene or 0, 25, 50, and 100 nM miR122 by the lipofectamine method. At 48h post-transfection, cell lysates were collected, and WB was performed with the indicated antibodies. Bottom panel in Fig 7B represented dose-dependent expression of ACSL4, GNMT, and IFI17 from left to right.

It is very well established that HIV-1 co-infection up-regulates HCV replication [7–9]. Inasmuch as the ER distribution of these proteins and differential expression of these genes within the liver disease context is stage-specific in co-infection, the proteins could play key roles in regulation of HCV replication. To elucidate the role of the genes in HCV replication, different amounts of each gene were transfected into APC140, and changes in expression of Renillar luciferase (RLuc) in the replicon were determined, since like other subgenomic replicons, APC140 harboring JFH1 strain of HCV replicates to very high levels, compared with the indolent HCV replication in cell culture, while recapitulating the intracellular steps of the HCV replication cycles in Huh7.5.1 [91–93]. Our Western blot analysis showed that the amount of each protein in APC140 increased in parallel with the increasing dose of the expressing plasmids, while the amount of β-actin was constant (Fig 7A, lower panel of each bar graph), indicating that the transfected gene was expressed in a dose-dependent fashion. Expression of *ACSL4* significantly enhanced RLuc activity, while nominal increases of the replicon expression were observed with changes to the amount of *miR122* (Fig 7A), whose increase was not comparable with *ACSL4*. In contrast, *GNMT* and *IFI27* inhibited HCV replication, lowering RLuc activity (Fig 7A), indicating that these two genes are negative regulators for HCV replication. Consistent with these data, expression of ACSL4 in Huh7.5.1 transfected with the full-length genomic HCV RNA enhanced expression of NS5A, while GNMT and IFI27 inhibited replicability of HCV, reducing the amount of NS5A in a dose-dependent manner (Fig 7B), even taking into consideration of excessive loading of GNMT (pC3 with GNMT). Transfection of miR122 induced nominal increase of NS5A expression (Fig 7B). Collectively, these data indicated that the four genes modulated liver disease progression by disparate regulation of HCV replication in the ER, thus also modulating HCV viremia.

### Effect of HIV-1 and its viral proteins on HCV replication

**Fig 8:**
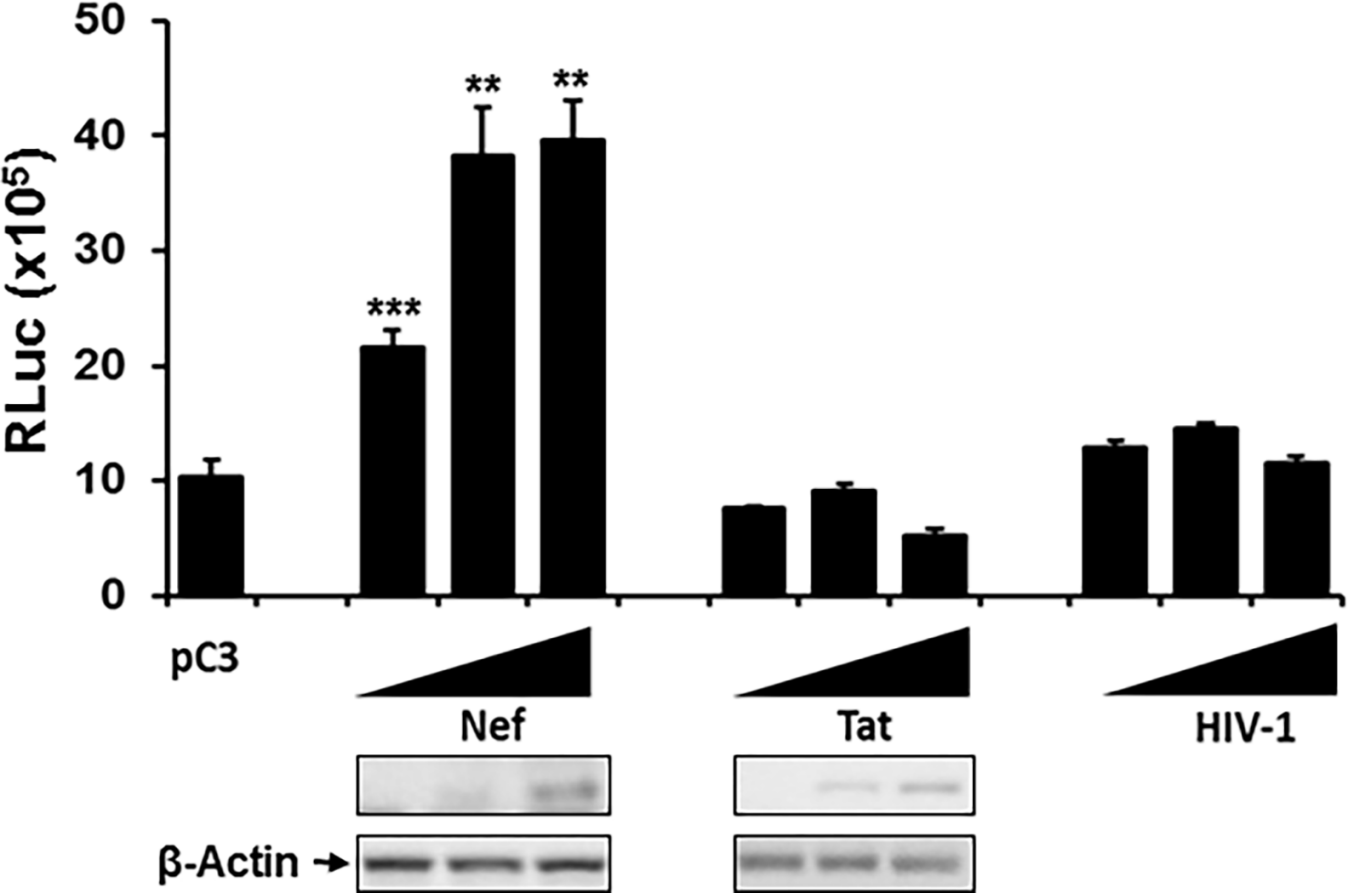
Evaluation of HIV-1 viral elements on the replicon expression. APC140 was transfected with 0.25, 0.5, and 1 μg of nef or 0.5, 1, and 2 μg of tat-expressing plasmid or treated with the purified HIV-1 corresponding to 25, 50, and 100 K cpm reverse transcriptase activity, and at 48 h post-transfection or treatment, cell lysates were prepared from the transfected cells, as described above. Renillar luciferase activity was measured, and WB was performed with anti-Myc Ab for Nef and Tat.

Hepatocytes were neither susceptible nor permissive for HIV-1 in our work—despite controversy in the literature on the replication potential of HIV-1 in hepatocytes [12–17, 19–21]—suggesting that HIV-mediated dysfunction of hepatocellular biology is not due to direct infection of HIV-1 into hepatocytes but to direct or indirect, intra- or extra-cellular interactions of HCV-infected hepatocytes with specific HIV-1 viral proteins. Since HIV-1 Env, Tat, and Nef among HIV viral proteins are the most well-known proteins that can transit from HIV-infected cells to the target hepatocytes via shedding or virions [14, 15], diffusion [94], and conduit/exosomes [23, 27–30], respectively, we first investigated the impact of HIV-1 Env on HCV replication. To this end, we treated APC140 with different amounts of Env embedded virions, such as VSV-Env, HIV-1, or purified HIV-1—not with the synthetic Env, insofar as Env on the surface of the virions maintains its native trimer of gp120, which is critical for interaction with the receptor and co-receptor molecules for virus entry [95–97]—and changes in the RLuc reading from APC140 were measured. Our data showed that a negligible or nominal increase in RLuc was detected, when the replicon cells were treated with different inocula of pseudotype HIV-1 (VSV-Env) or with HIV-1 (data not shown). Similarly, treatment of APC140 with the purified HIV-1 did not induce discernible increases in HCV replication (Fig 8). Expression of HIV-1 *tat* itself was not sufficient for up-regulation of HCV replication, even with appreciable expression of the protein (Fig 8), which is reasonable, given that Tat is a nuclear protein [83, 84], whereas HCV replication takes place in the ER. In contrast, expression of *nef* dramatically increased replicability of HCV in a dose-dependent manner, as shown in Fig 8, which is consistent with our previous observation [23]. Taken together, these data indicated that HIV-1 Nef, and neither Env/HIV-1 nor Tat, is the most critical determinant for co-infection-induced enhancement of HCV replication and thereby exacerbation of HCV-mediated liver malady.

### Impact of HIV-1 viral proteins on expression of the four cellular genes

**Fig 9:**
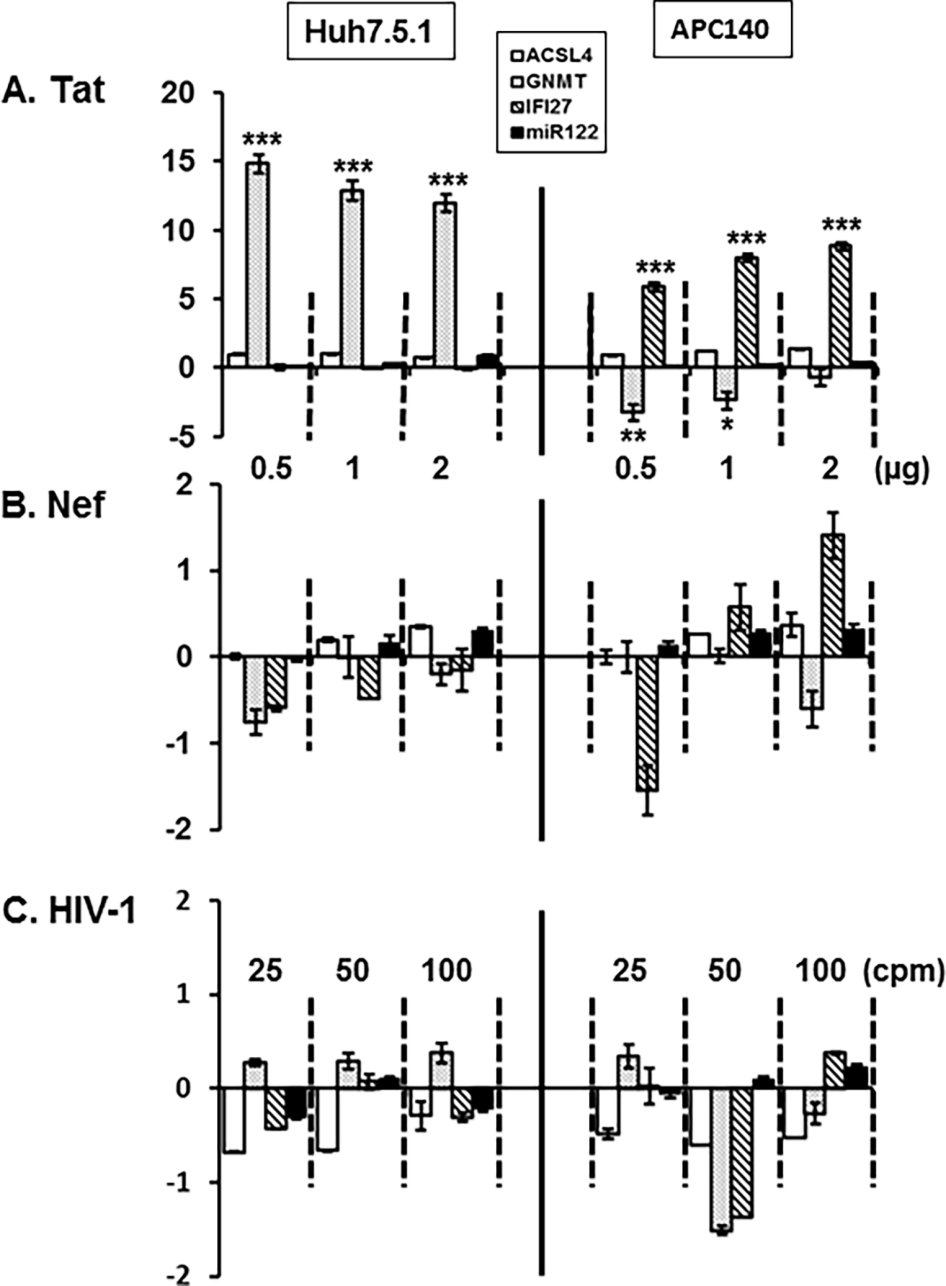
Effect of HIV-1 viral proteins on expression of the cellular genes. Huh7.5.1 and APC120 were transfected with 0.5, 1, and 2 μg of *nef*- or *tat*-expressing plasmid or treated with the purified HIV-1 corresponding to 25, 50, and 100 K cpm reverse transcriptase activity, and at 48 h post-transfection or treatment, RNAs isolated from the transfected or treated cells were quantified by the qRT-PCR Assay (Bio-Rad, Hercules, CA), as triplicate, using the specific primer pairs of each gene. The β-Actin house-keeping gene was used as an internal control for qRT-PCR, and *U6-2* was employed as an internal control for *miR122*. Melting curve analysis was done to confirm the specificity and purity of the amplified product.

Acceleration of liver disease progression by co-infection could be achieved not only by enhancement of HCV viremia but also by dysregulation of hepatocellular genes critical for the liver disease by the transferred HIV and/or its viral proteins from HIV-1-infected cells. Hence, we explored the impact of Env, Nef, and Tat on expression of *ACSL4*, *GNMT*, *IFI27*, and *miR122* in Huh7.5.1 and APC140. Here, our major results were: First, only HIV-1 Tat significantly altered expression of the genes (Fig 9A), although Nef increased expression of *IFI27* in a dose-dependent manner (Fig 9B), and Env at moderated concentration inhibited *GNMT* and *IFI27* (Fig 9C), which is reasonable, based on the well-established molecular function of Tat. That is, among these elements Tat is the only protein located in the nucleus and is known to trans-activate various cellular genes [83, 84]. Second, expression patterns of those cellular genes under Tat in Huh7.5.1 are quite distinct from those of the genes in APC140, indicating that HCV regulatory proteins expressed from the subgenomic replicon are also involved in modulation of the gene expression. To elaborate, transcription of the *GNMT* gene was augmented by approximately 15 fold in Huh7.5.1 (Fig 9, dotted bar), but was reduced in the presence of regulatory proteins of HCV (APC140) (Fig 9), mimicking reduction of the gene expression at different stages of liver diseases by mono- and co-infection (Fig 4). Changes in expression of *IFI27* were negligible in Huh7.5.1, whereas the level was dramatically increased up to 7 fold by Tat in APC140 (Fig 9, stripped bar). Similarly, subtle reduction in the level of *ACSL4* in Huh7.5.1, but a steady increase of the gene in APC140 (Fig 9, open bar) and a nominal increase in Huh7.5.1 but little or no expression of *miR122* in APC140 (Fig 9, closed bar), were also observed. These data together demonstrated that HIV-1 Tat among the tested HIV-1 viral proteins was the most critical element in regulating the selected cellular gene expression and that HIV-1, together with HCV viral proteins, via co-infection could exert dramatic changes in the expression of cellular genes integral to liver complications.

### Combinatorial effect of HIV-1 viral and the cellular genes on HCV replication

**Fig 10:**
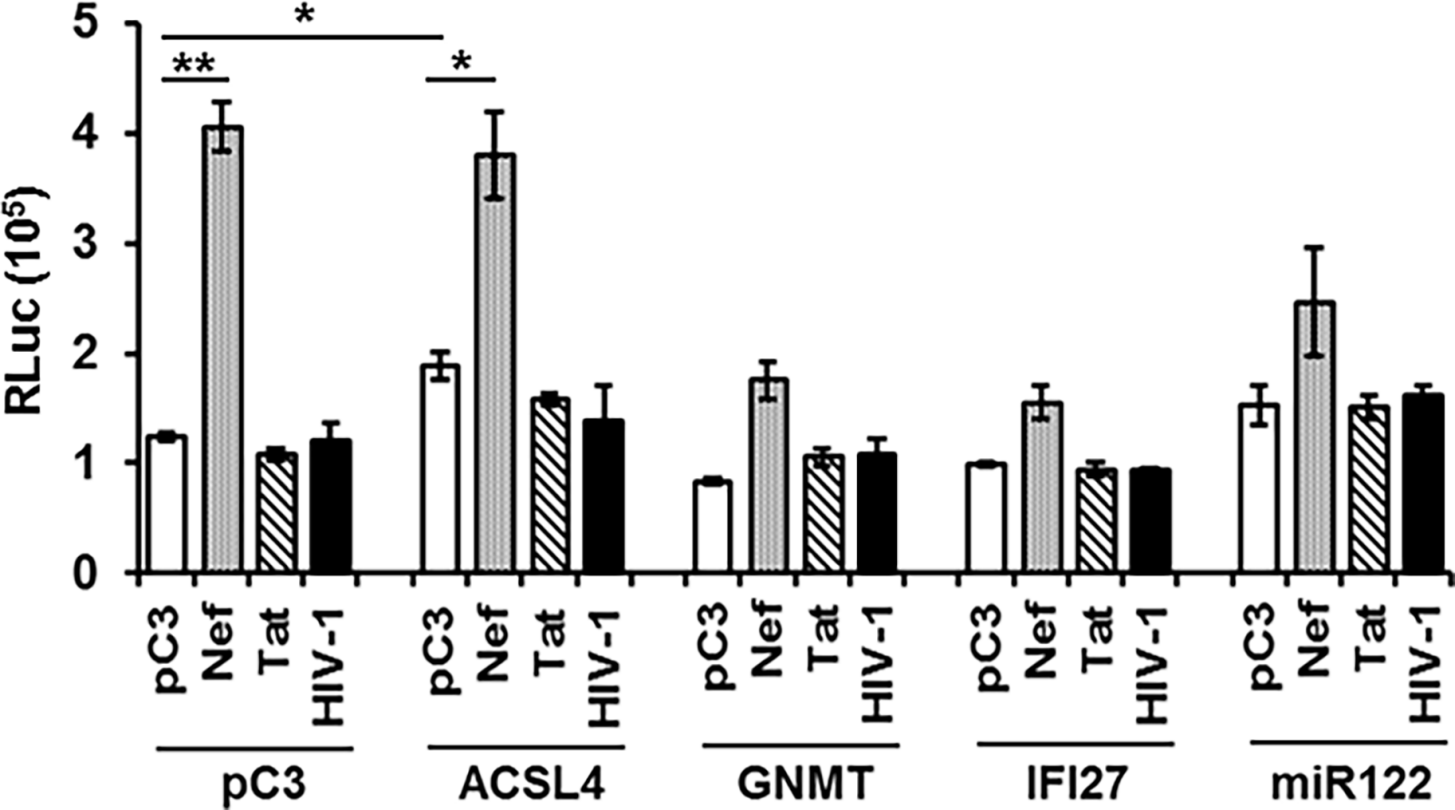
Combinatorial effects of viral and cellular genes on replicon expression. APC120 was transfected with 0.5 μg of the indicated plasmid expressing its corresponding cellular protein or 25 nM *miR122* and with 0.5 μg of nef- or tat-expressing plasmid, or treated with the purified HIV-1 corresponding to 25,000 cpm reverse transcriptase activity. At 48 h post-transfection or treatment, cell lysates were prepared from the transfected cells, as described above, and Renillar luciferase activity was measured as triplicate.

Since ACSL4 and miR122 up-regulated, while GNMT and IFI27 impaired replicon expression (Fig 7), and HIV-1 Nef and Tat altered the level of replicon expression (Fig 8) and cellular gene expression (Fig 9), respectively, we investigated combinatorial impacts of viral and cellular genes on the replicon expression. Consistent with the data in Fig 8, only Nef, but not Tat or Env, enhanced replicon expression (Fig 10, pC3 bracket). ACSL4 and miR122 increased, whereas GNMT and IFI27 reduced replicon expression in the absence of HIV-1 viral proteins (Fig 10, each gene with pC3), which also showed a consistent pattern of changes in replicon expression by action of the cellular genes, as shown in Fig 7. Interestingly, Nef, a strongly positive HIV-1 regulator for the replicon expression, elevated the cellular gene-mediated replicon expression, but this did not reach the level of Nef-only induced enhancement of replicon expression, even with ACSL4 or miR122 (Fig 10). In view of the negative role of GNMT and IFI27 in the replicon expression (Fig 7), it is reasonable to expect moderate increases of luciferase activity even with Nef (Fig 10). Co-expression of HIV-1 Tat and of the individual genes did not show discernible changes in the gene-triggered HCV replicability (Fig 10), even if Tat were the main determinant for expression of these cellular genes. These data suggested reciprocal actions between viral and cellular genes in modulation of HCV replication.

## Discussion

Our microarray data identified numerous hepatocellular genes which were expressed in a liver disease stage-specific manner by co-infection, and we have chosen 3 genes (*ACSL4*, *GNMT*, *IFI27*) and 1 miRNA (*mir122*), whose expression was consistently either up- or down-regulated in a disease stage-specific manner in all 3 co-infected patients’ subjects’ liver. Our study further showed that Tat impacted most the expression of the indicated cellular genes, while Nef played a cardinal role in regulation of replicon expression and thus HCV replication. Collectively, these data demonstrated that HIV-1 viral proteins could exacerbate liver disease in the co-infected patients by disparate molecular mechanisms—dysregulating HCV replication directly and/or indirectly modulating expression of hepatocellular genes critical for the liver disease progression. These data also provide vital insights into development of liver disease stage-specific biomarkers for diagnosis and prognosis of liver diseases, based on the stage-specifically expressing hepatocellular genes in the co-infected patients.

*ACSL4*, *GNMT*, *IFI27*, and *miR122* are known or expected to play a key role in the clinical courses of HCV-mediated liver diseases by regulating HCV replication and thereby hepatocellular carcinoma. To elaborate, *ACSL4*, the only differentially expressed gene among several other of its isotype genes by co-infection, is distinguished by its preference for arachidonic acid so as to alter the amount of intracellular prostaglandin, phosphatidylinositol, and so on [98], which is functionally distinct from the isotype genes [41]. Investigation of the corroborative changes of those metabolites along with the liver disease stages will therefore bring insights into the utility of the molecules as markers for diagnosis and prognosis of co-infection mediated liver disease advancement. Likewise, *GNMT*, the most abundant methyltransferase in the liver [55], is associated with fatty liver disease which accompanies hepatic methionine deficiency and homocysteine elevation, resulting mainly from impaired homocysteine demethylation and aberrant reactions of methyltransferase [57–59]; that is, changes in methionine and homocysteine levels could be clinical milestones in progression of co-infected liver malady. *IFI27* is involved in the IFN pathway [60–66] and was found to be up-regulated in HCC [67–69], indicating that IFI27/IFN could be a valuable indicator of co-infection-associated immune changes. Further, expression of *miR122* is downregulated in HCC, suggesting that it can serve as a biomarker for liver cancer [85, 99–101]. Significance and molecular mechanisms of HIV-1 co-infection-mediated down-regulation of miR122 (Fig 4) with respect to HCV replication and acceleration of liver disease progression are unknown. It is speculated that, in light of the previous reports wherein HCV RNA functionally sequesters miR122 and thus reduces its bioavailability [102] [103] to maintain stoichiometry between HCV RNA and miR122 for HCV replication [102], reduction of the level of intracellular miR122 by co-infection could contribute to regulating bioavailability of the miRNA for HCV replication. Since it is expressed differentially in a liver disease stage-specific manner by co-infection, it could also be developed as a biomarker for diagnosis and prognosis of co-infection-mediated hepatic disease progression. Thus, the elucidation of whether production of the indicated metabolites is concomitant with the differential expression of the genes in a liver disease stage-specific manner will clarify the molecular processes of HIV co-infected, HCV-mediated hepatic disease, and will augment development of molecular biomarkers for stage-specific hepatic disease acceleration by co-infection.

A significant portion of each protein produced from the differentially expressed genes is localized to ER (Fig 6) where HCV replication takes place, which raises the possibility of involvement of the proteins in regulating the HCV life cycle. In fact, expression of ACSL4, GNMT, and IFI27 has been found in the ER (Fig 6) and altered replicon expression (Fig 7). A remarkable observation for GNMT is that puncta in Huh7.5.1 vanished upon expression of HCV regulatory proteins in APC140 (Fig 6), suggesting that HCV viral proteins altered the subcellular distribution pattern of the protein. The implications of this alteration with respect to HCV life cycle require further study.

Our investigations reveal HIV viral determinants responsible for the observed differential expression of the genes and regulation HCV replication. Tat is the most critical HIV-1 viral element in regulation of transcription to generate the candidate cellular genes (Fig 9), which is expected from the well-known function in trans-activation of the expression of various genes, and in subcellular localization of the protein in the nucleus (93,94). Further, Tat-triggered differential expression of these genes in the presence (APC140) and absence (Huh7.5.1) of HCV viral proteins was dramatic, especially for GNMT and IFI27, substantiating the significance of co-operative action between HIV-1 and HCV viral proteins in regulating the cellular genes in the co-infection-mediated pathobiology of hepatocytes. Even if Tat regulated the hepatocellular gene expression to modulate replicon expression, interestingly, the expression of Tat alone (Fig 8), or of Tat with the above four genes (Fig 10) did not trigger discernible changes in replicon expression. The negligible modulation of HCV replication by Tat (Fig 8), which regulates hepatocellular gene expression significantly (Fig 9), could be due to the regulation of expression of numerous hepatocellular genes involved in not only positive, but also negative, regulation of HCV replication, and thus the overall effect of Tat on HCV replication was intangible. Unlike Tat, Nef played a cardinal role in regulation of replicon expression (Fig 8). Further, combinatorial expression of Nef with each cellular gene yielded a higher level of replicon expression than the individual gene did, but this did not attain the level of expression seen with Nef alone, even in conjunction with ACSL4 that also functioned as a positive element for the replicon expression (Fig 10). However, we do not know molecular mechanisms whereby co-expression of Nef and positively acting cellular genes, such as ACSL4, for HCV replication lack synergism or additive effects, even if independent expression of these genes increased HCV replication. Further investigation behind this observation is imperative to understand co-infection-mediated acceleration of liver disease progression. Taken together, these results showed that HIV-1 viral proteins can exacerbate liver pathology in the co-infected patients by disparate molecular mechanisms. Moreover, elucidation of the molecular mechanisms for how the viral and cellular proteins independently from, or cooperatively with, HCV contribute to the observed changes could raise insight into the molecular processes for HIV co-infected, HCV-mediated hepatic disease, and will promote the generation of molecular biomarkers for stage-specific liver disease acceleration by co-infection.

Finally, we recognize that the three liver specimens from each stage of HCV-mono- and HIV-1/HCV-co-infected patients may not be numerically optimal to serve more systematic conclusions on the molecular significance of the differential expression of the cellular genes. However, we believe that our extensive computational and molecular analyses with the longitudinal, not cross-sectional samples, wherein each sample could function as a control for another group of samples, do combine to indicate the importance of our described genes in stage-specific singly and dual-infected viral liver disease. We consequently are committed to expanding our biostatistical studies with the ever improving technology and increasing sample sizes. Our reported experiments support future study for mechanistic insights and retrieval of clinically germane diagnostic and prognostic molecular candidates in HCV- and HCV/HIV-1 viral hepatic disease.
